# Caffeic Acid Mitigates L-Methionine-Induced Impairment of Hippocampal Neural Stem Cells Through Anti-Apoptotic Signaling in Adult Rats

**DOI:** 10.3390/biology15181615

**Published:** 2026-09-14

**Authors:** Oabnithi Dornlakorn, Nataya Sritawan, Ram Prajit, Apiwat Sirichoat, Peter Wigmore, Jariya Umka Welbat

**Affiliations:** 1 Department of Anatomy, Faculty of Medicine, Khon Kaen University, Khon Kaen 40002, Thailand; oabnithiatlakorn@kkumail.com (O.D.); natasr@kku.ac.th (N.S.); rampra@kku.ac.th (R.P.); apiwsi@kku.ac.th (A.S.); 2 Neurogenesis Research Group, Faculty of Medicine, Khon Kaen University, Khon Kaen 40002, Thailand; 3 School of Life Sciences, Medical School, Queen’s Medical Centre, The University of Nottingham, Nottingham NG7 2RD, UK; peter.wigmore@nottingham.ac.uk

**Keywords:** caffeic acid, L-methionine, neural stem cells, apoptosis

## Abstract

Chronic administration of L-methionine (L-met) induces hyperhomocysteinemia, which triggers oxidative stress, impairs hippocampal neurogenesis and ultimately causes cognitive decline. Caffeic acid (CA) has been reported to exert neuroprotective effects through its antioxidant properties, attenuating oxidative damage in the brain, promoting hippocampal neurogenesis, and improving cognitive function. This study evaluated the neuroprotective efficacy of CA against L-met-mediated hyperhomocysteinemia and associated hippocampal neurotoxicity in a rat model. Twenty-four male Sprague-Dawley rats were randomly divided into four groups (n = 6/group): Vehicle, L-met (1.7 g/kg), CA (40 mg/kg), and CA + L-met. Treatments were administered orally once daily for 28 days. Immunofluorescence analysis revealed that CA effectively prevented L-met-induced depletion of nestin-positive cells. Structural assessments further confirmed that CA counteracted tissue atrophy, preserving the anatomical volumes of both the dentate gyrus (DG) and granule cell layer (GCL). Furthermore, CA attenuated the L-met-induced downregulation of the neurogenesis-related proteins nestin and Notch-1 in the hippocampus. Notably, CA also modulated apoptosis-related protein expression by reducing Bax and Caspase-3 levels while upregulating Bcl-2 expression. In conclusion, CA was associated with protection against L-met-induced alterations in hippocampal neurogenesis- and apoptosis-related protein expression, as well as preservation of hippocampal architecture, in this experimental model.

## Simple Summary

This study evaluated the neuroprotective efficacy of caffeic acid (CA) against chronic L-methionine (L-met)-induced hyperhomocysteinemia and hippocampal neurotoxicity in rats. Twenty-four male Sprague-Dawley rats (n = 6/group) were orally administered vehicle, L-met (1.7 g/kg), CA (40 mg/kg), or CA + L-met for 28 consecutive days. The findings demonstrate that CA administration was associated with preserved hippocampal architecture, characterized by an absence of tissue atrophy and maintenance of the anatomical volumes of both the dentate gyrus and the granule cell layer. Immunofluorescence and molecular analyses demonstrated that CA counteracted L-met-induced neurotoxicity by preventing the depletion of nestin-positive cells and attenuating the downregulation of nestin and Notch-1, key proteins involved in neurogenesis. Furthermore, CA modulated apoptosis-related protein expression by downregulating the pro-apoptotic factors Bax and Caspase-3 and upregulating the anti-apoptotic protein Bcl-2. These findings suggest that CA protects against L-met-induced hippocampal neurotoxicity by preserving neurogenesis-related protein expression, attenuating apoptosis-related protein alterations, and maintaining structural integrity.

## 1. Introduction

As the global population ages at an accelerating rate, neurocognitive disorders, particularly dementia, have become a paramount public health challenge [1]. These disorders are characterized by a progressive decline in cognitive function, encompassing deficits in executive function, memory, and language [2]. The pathogenesis of dementia is increasingly recognized as a consequence of systemic metabolic dysregulation and the accumulation of neurotoxic metabolites, rather than a mere reflection of chronological aging [3]. A key component of this neurodegenerative cascade is neuronal apoptosis, a form of programmed cell death triggered by pathological elevation of endogenous metabolites—most notably homocysteine (Hcy), which precedes and promotes the deposition of amyloid-beta and phosphorylated tau [4]. These biochemical insults induce severe cellular stress, characterized by excitotoxicity and a profound failure of mitochondrial bioenergetics [5].

L-methionine (L-met), the essential amino acid precursor of the universal methyl donor S-adenosylmethionine (SAMe), must be obtained through diet [6]. To induce mild hyperhomocysteinemia (HHcy), a 1.7 g/kg dose of L-met was administered, which reliably elevates serum Hcy levels above 15 µmol/L and provides an established model for neurotoxicity assessments [7]. The methionine cycle is essential for DNA methylation, phospholipid synthesis, and neurotransmitter metabolism [8]. Under physiological conditions, L-met levels are regulated via the remethylation (requiring vitamin B_12_ and folate) and transsulfuration (mediated by CBS and vitamin B_6_) pathways [9]. Moreover, when L-met dietary intake exceeds the capacity of the remethylation and transsulfuration pathways or in the presence of vitamin B deficiencies, Hcy accumulates, leading to protein misfolding via Hcy thiolactone reactivity [10]. Hcy thiolactone reacts with protein lysine residues, causing misfolding and functional impairment [11]. Furthermore, HHcy—primarily induced by excessive accumulation—exerts potent neurotoxic effects through a multifaceted “triple hit” mechanism comprising excitotoxicity, oxidative stress, and epigenetic dysregulation [12]. Mechanistically, Hcy activates N-methyl-D-aspartate (NMDA) receptors, causing pathological calcium influx that initiates apoptotic signaling and neuronal injury [13]. This process is further exacerbated by mitochondrial dysfunction and the accumulation of reactive oxygen species (ROS), which together compromise genomic stability in post-mitotic neurons [14]. Moreover, chronic administration of 1.7 g/kg L-met induces HHcy in male rats, precipitating memory deficits via suppressed hippocampal neurogenesis and reduced dentate gyrus cell proliferation [15].

Emerging evidence suggests that caffeic acid (CA), a ubiquitous hydroxycinnamic acid found in coffee and fruits, exerts robust neuroprotective effects against HHcy-induced neurotoxicity [16]. Beyond its dietary role as a phenolic compound, CA exhibits potent antioxidant and anti-inflammatory properties, attenuating Hcy-mediated damage by neutralizing ROS and lipid peroxidation and restoring endogenous antioxidant enzyme activity [17]. Crucially, CA has been shown to downregulate pro-apoptotic cascades, specifically caspase-3 activation, and upregulate the expression of brain-derived neurotrophic factor (BDNF) [7]. These interventions enhance neuronal survival and proliferation within the hippocampal dentate gyrus and concurrently ameliorate memory deficits associated with L-met-induced metabolic stress [18]. Consequently, this study aimed to investigate whether CA treatment could mitigate the adverse effects of chronic L-met administration on hippocampal integrity, with a focus on apoptotic signaling and impaired neurogenesis.

## 2. Materials and Methods

### 2.1. Animal Experiment Procedure

**Figure 1:**
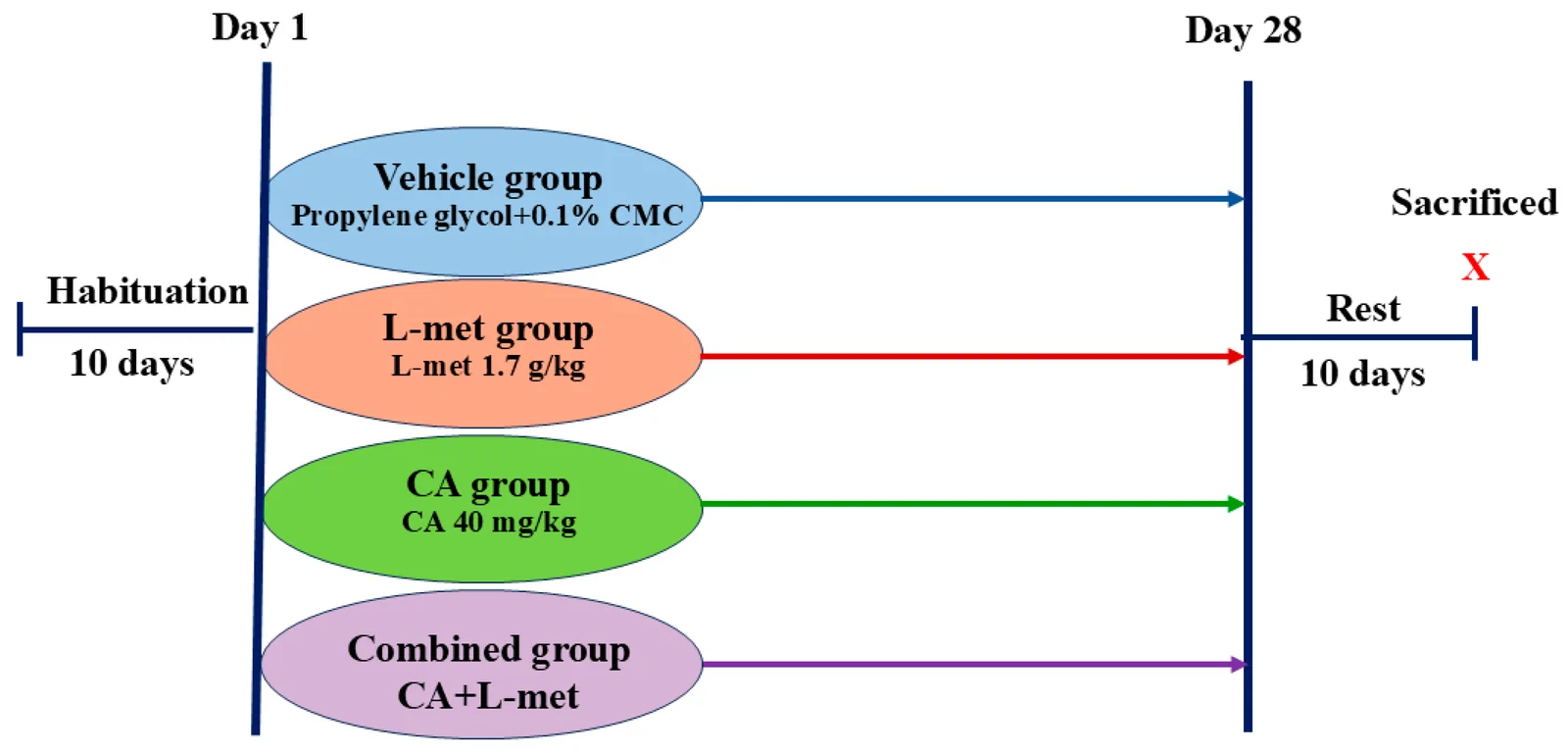
Timeline of drug administration. Rats received daily oral gavage of vehicle, L-met (1.7 g/kg), CA (40 mg/kg), or combined CA + L-met for 28 days. All animals were sacrificed on day 38 for tissue collection.

The experimental design and treatment protocol are illustrated in Figure 1. A sample size of six animals per group was chosen based on previous studies using similar animal models [15]. Twenty-four male Sprague-Dawley rats (200–220 g, 4–5 weeks old) were obtained from Nomura Siam International Co., Ltd. (Bangkok, Thailand), and acclimatized for seven days before experimentation. As outlined in Figure 1, following the acclimatization period, the animals were housed in stainless steel cages measuring 28 × 47 × 18 cm, maintained at a controlled room temperature and a 12-h light/dark cycle, and provided with free access to standard chow and water. Rats were monitored daily for general health, body weight, and signs of distress throughout the experimental period. No environmental enrichment was provided, and no adverse events occurred; predefined humane endpoint criteria were not met at any point during the study. All procedures were approved by the Khon Kaen University Ethics Committee in Animal Research (Ref. No. AICUC-KKU-87/65). L-met and CA were obtained from Sigma-Aldrich Chemical Company (St. Louis, MO, USA). As summarized in the experimental timeline (Figure 1), rats were randomly allocated into four groups for a 4-week oral gavage regimen: (1) the vehicle control group, receiving propylene glycol and 0.1% *w/v* carboxymethyl cellulose (CMC); (2) the L-met group, receiving L-met (1.7 g/kg/day) dissolved in 0.1% *w/v* CMC; (3) the CA group, receiving CA (40 mg/kg/day) dissolved in propylene glycol; and (4) the combined (CA + L-met) group, receiving both CA and L-Met at the doses specified above. Animals were obtained from the same cohort and maintained under identical environmental and husbandry conditions as in our previous study [7], which confirmed that L-met administration significantly elevated serum Hcy levels (Figure 1).

### 2.2. Immunofluorescence Assessment

Following fixation in 4% paraformaldehyde (PFA; Sigma-Aldrich, Inc., St. Louis, MO, USA), rat brains were serially sectioned coronally at a thickness of 40 µm using a cryostat (Series HM550, Microm International, Walldorf, Germany). The sections were stored in a cryoprotective buffer at 4 °C until use. For nestin immunohistochemistry, every eighth section spanning the length of the dentate gyrus (DG) was selected using a systematic random sampling approach, yielding nine sections per brain. The selected sections were washed with phosphate-buffered saline (PBS) and permeabilized with 0.05% Triton X-100. Subsequently, the sections were incubated overnight at 4 °C with a primary anti-nestin antibody (1:400). The following day, the sections were incubated at room temperature for 60 min with an Alexa Fluor 488 rabbit anti-mouse IgG secondary antibody (1:500; Invitrogen, Carlsbad, CA, USA), followed by counterstaining with propidium iodide (1:6000; Sigma-Aldrich, Inc., St. Louis, USA) for 30 s. Finally, the sections were mounted with glycerol, and nestin-positive cells were visualized under a fluorescence microscope [19].

For the volumetric assessment of the DG and granule cell layer (GCL), a separate set of 20 µm thick coronal sections was collected. Using a systematic sampling design, every 15th section along the rostro-caudal axis of the DG was selected, yielding nine sections per brain. The sections were stained with DAPI for nuclear counterstaining and imaged under a 4× objective lens using a fluorescence microscope. The cross-sectional areas of the DG and GCL were quantified using ImageJ software (version 1.53a; National Institutes of Health, Bethesda, MD, USA). The total volume for each region was calculated by multiplying the sum of the measured areas by the section interval factor (15) and the section thickness (20 µm) [20].

### 2.3. Western Blotting Analysis

Hippocampal tissues were homogenized on ice using a manual pestle in a lysis buffer containing 20 mM Tris (Sigma-Aldrich, St. Louis, MO, USA; pH7.6), 1 mM EGTA (Sigma-Aldrich, St. Louis, MO, USA), 320 mM sucrose (Sigma-Aldrich, St. Louis, MO, USA), 0.1% Triton X-100 (Sigma-Aldrich, St. Louis, MO, USA), 1 mM NaF (Sigma-Aldrich, St. Louis, MO, USA),10 mM glycerophosphate (Sigma-Aldrich, St. Louis, MO, USA), and protease inhibitor cocktail (Roche, Basal, Switzerland), to achieve a final tissue concentration of 100 mg/mL. Homogenates were centrifuged at 13,000 rpm (4 °C, 10 min), and the supernatants were collected. Total protein concentrations were quantified by direct UV absorbance at 280 nm (A280) using a NanoDrop spectrophotometer (Thermo Fisher Scientific, Waltham, MA, USA). For immunoblotting, 30 µg of protein per lane was separated by sodium dodecyl sulfate–polyacrylamide gel electrophoresis (SDS-PAGE) (Bio-Rad Laboratories, Hercules, CA, USA) using either 10% resolving gels (for Nestin and Notch1) or 12% resolving gels (for Bax, Bcl-2, and caspase-3). The separated proteins were subsequently transferred onto nitrocellulose membranes (GE Healthcare Life Sciences, Freiburg, Germany). Membranes were blocked with 5% non-fat milk (or BSA) (Sigma-Aldrich, St. Louis, MO, USA) in TBS-T) (Bio-Rad Laboratories, Hercules, CA, USA) for 1 h at room temperature to prevent non-specific binding, then incubated overnight at 4 °C with the following primary antibodies: anti-Nestin (1:1000; MAB353, Merck, Darmstadt, Germany), anti-Notch1 (1:100; sc-6014 Santa Cruz Biotechnology, Dallas, TX, USA), anti-Bax (1:1000; ab53154, Abcam, Cambridge, UK), anti-Bcl-2 (1:2000; ab59348, Abcam, Cambridge, UK), anti-Caspase-3 (1:2000; sc-7272, Santa Cruz Biotechnology, TX, USA), and anti-GAPDH (1:20,000; ab8245, Abcam, Cambridge, UK) as a housekeeping control. Following primary incubation, the membranes were incubated with appropriate horseradish peroxidase-conjugated anti-rabbit or anti-mouse secondary antibodies (1:2000; P0447, DAKO, Glostrup, Denmark) for 60 min at room temperature. Protein bands were visualized using an enhanced chemiluminescence (ECL) detection system (GE Healthcare, Buckinghamshire, UK). The relative optical density of the immunoreactive bands was quantified using ImageJ software (version 1.53a; National Institutes of Health, Bethesda, MD, USA) and normalized to GAPDH. All experimental assays were performed in triplicate.

### 2.4. Statistical Analysis

Statistical parameters were calculated using GraphPad Prism (version 7.04) and IBM SPSS Statistics (version 29). Data are expressed as mean ± SEM, and the threshold for statistical significance was set at *p* < 0.05. The normality of the data distribution was verified using the Kolmogorov–Smirnov and Shapiro–Wilk tests (*p* > 0.05). Differences in Nestin-immunofluorescence-positive cell counts, protein expression, and DG and GCL volumes were analyzed using one-way ANOVA. Statistical significance among groups was assessed by one-way ANOVA, followed by Bonferroni’s post hoc test for multiple comparisons.

## 3. Results

### 3.1. Effect of CA and L-Met on Nestin-Positive Cell Expression

**Figure 2:**
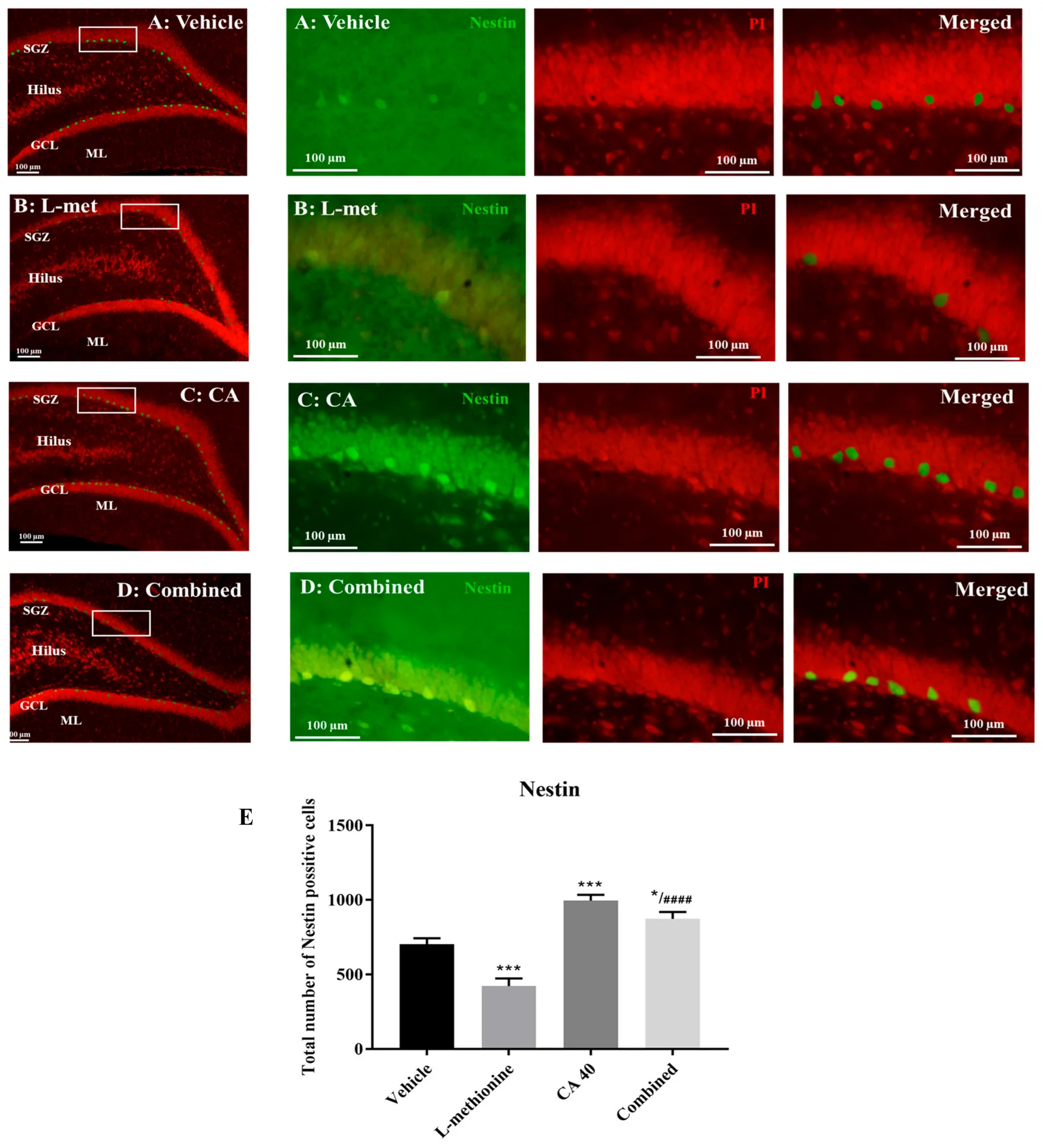
Cell expressing nestin (green) are shown in image (**A**) (vehicle group, (**B**): L-met group, (**C**): caffeic acid group, (**D**): Combined group, with propidium iodide (PI) (red) used as a counterstain. (**E**) The number of nestin-specific cells in the SGZ of the DG was quantified through cell counting. The results revealed significant differences in the L-met (*** *p* < 0.001), CA40 (*** *p* < 0.001), and combined (* *p* < 0.05) groups compared with the vehicle group. Furthermore, the combined group exhibited a significant difference compared to the L-met group (^####^ *p* < 0.0001). GCL: Granular cell layer; ML: Molecular layer; SGZ: Subgranular zone.

Quantitative assessment of nestin-positive cells (visualized by immunohistochemistry, as shown in Figure 2A–D) revealed a significant reduction in the L-Met group compared with the vehicle group (F (3, 20) = 31.23, R^2^ = 0.8241, *p* = 0.0007). Conversely, the group receiving CA alone showed a significantly greater number of nestin-positive cells compared with the vehicle group (F (3, 20) = 31.23, R^2^ = 0.8241, *p* = 0.0005). Notably, CA administration to the L-met group resulted in a significantly higher number of nestin-positive cells compared with both the vehicle and L-met groups (F (3, 20) = 31.23, R^2^ = 0.8241, *p* = 0.0406 and *p* < 0.0001, respectively). Quantification in the subgranular zone (SGZ) of the dentate gyrus (DG) (Figure 2E) confirmed these findings, indicating that CA not only rescues neural stem cells from L-met-induced depletion but also enhances nestin-positive cell populations beyond baseline observed in the vehicle group.

### 3.2. Effect of CA and L-Met on DG and GCL Volumetric Assessment

**Figure 3:**
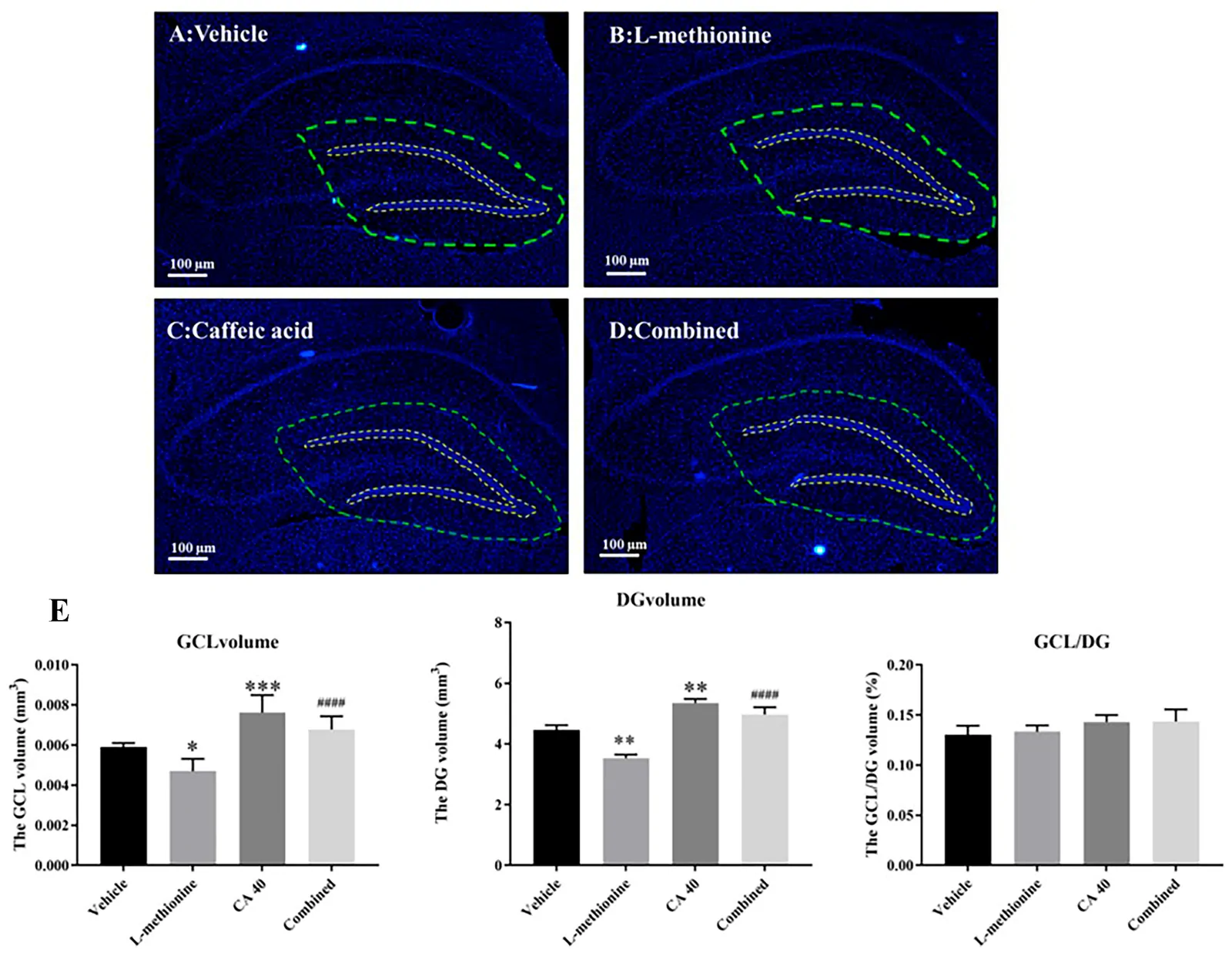
Hippocampal architecture, stained with DAPI (blue), is shown with a scale bar of 100 µm. Representative images are shown for the following groups: (**A**): vehicle group, (**B**): L-met group, (**C**): caffeic acid group, (**D**): combined group (**A**–**D**). The DG and GCL volumes are presented as a relative percentage, with *p*-values indicating significant differences: ** *p* < 0.01, *** *p* < 0.001, and * *p* < 0.05, compared with the control group. Additionally, the combined group exhibited a significant difference compared with the L-met group (^####^ *p* < 0.0001) (**E**).

The volumetric assessment of the DG and its GCL revealed significant structural changes across the experimental groups. Chronic L-met administration resulted in a significant reduction in both total DG (F (3, 20) = 21.68, R^2^ = 0.7648, *p* = 0.0027) and GCL volume (F (3, 20) = 23.27, R^2^ = 0.7773, *p* = 0.0110) compared with the vehicle group. Conversely, the CA treatment group exhibited a significant increase in both DG (F (3, 20) = 21.68, R^2^ = 0.7648, *p* = 0.0042) and GCL (F (3, 20) = 23.27, R^2^ = 0.7773, *p* = 0.0004) volumes compared with the vehicle group. Importantly, in the group receiving combined treatment (L-met + CA), DG and GCL volumes were preserved, showing no significant differences from the vehicle group while remaining significantly higher than those in the L-met group (DG: F (3, 20) = 21.68, R^2^ = 0.7648, *p* < 0.0001); GCL: F (3, 20) = 23.27, R^2^ = 0.7773, *p* < 0.0001). Furthermore, although L-Met treatment was associated with reduced DG and GCL volumes, the DG/GCL volume ratio remained consistent among all experimental groups, suggesting that L-Met-induced volume changes affected the DG and GCL proportionally, independent of CA treatment (Figure 3).

### 3.3. Effects of CA on Neural Stem Cell Maintenance: Nestin and Notch1 Expression

**Figure 4:**
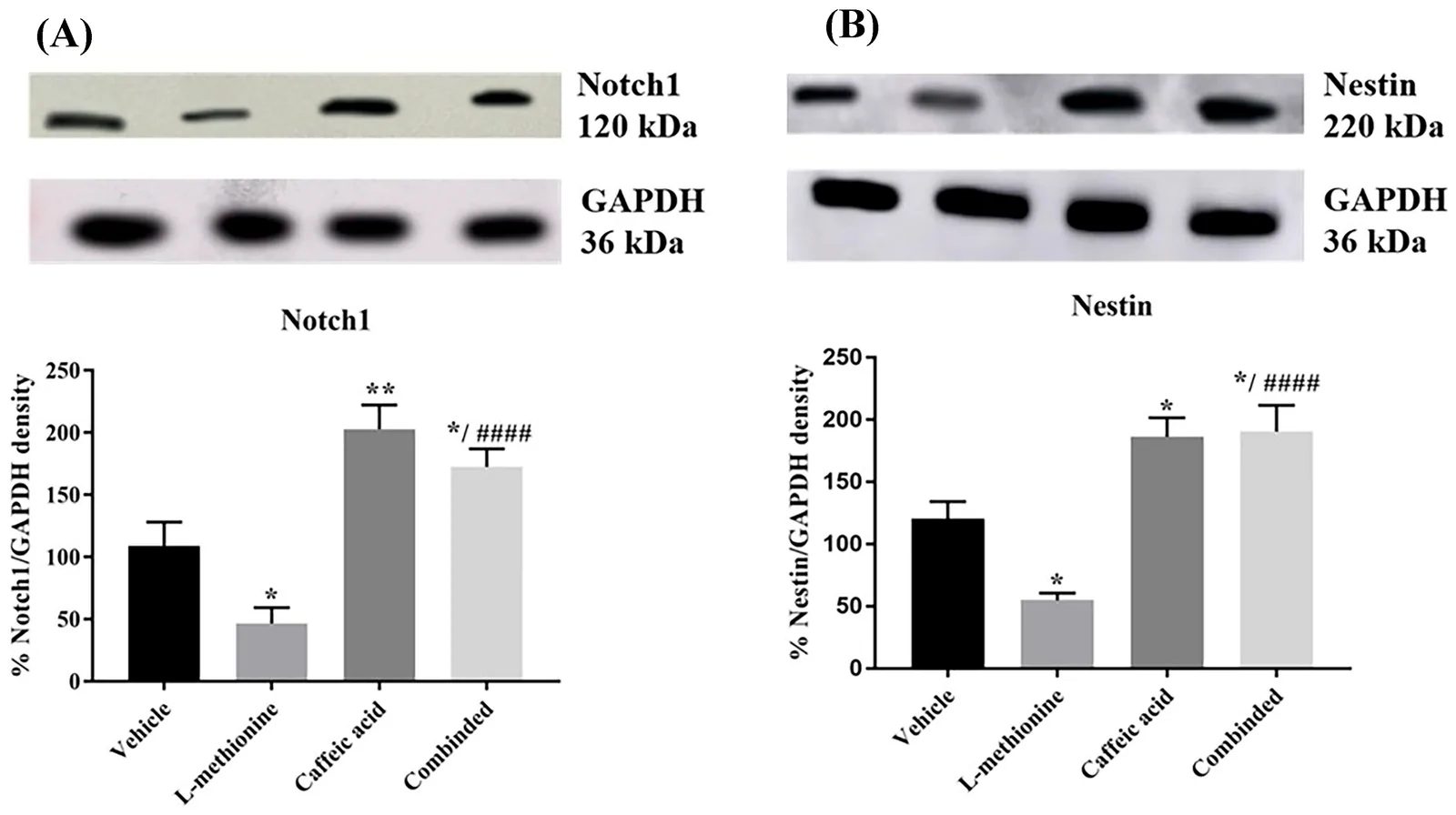
Protein expression levels in hippocampal tissue. (**A**) Representative Western blot bands and quantitative analysis of Notch1 expression. (**B**) Representative Western Blot bands and quantitative analysis of nestin expression. Protein levels were normalized and compared relative to both the vehicle group (* *p* < 0.05, ** *p* < 0.01) and the L-met group (^####^ *p* < 0.0001).

To investigate the effect of CA on neural stem cell status in the L-met-induced HHcy model, we evaluated the protein expression of nestin and Notch1. The administration of L-met resulted in a significant downregulation of Notch1 (F (3, 20) = 17.34, R^2^ = 0.7223, *p* = 0.0464) and nestin (F (3, 20) = 18.29, R^2^ = 0.7329, *p* = 0.0167) protein expression compared to the vehicle group, indicating an impairment in neural stem cell status. In contrast, CA administration significantly increased the expression of both Notch1 (F (3, 20) = 17.34, R^2^ = 0.7223, *p* = 0.0022) and nestin (F (3, 20) = 18.29, R^2^ = 0.7329, *p* = 0.0167) markers compared with the vehicle group. Crucially, the combined group (CA + L-met) showed significantly higher expression levels than the L-met group (Notch1: F (3, 20) = 17.34, R^2^ = 0.7223, *p* < 0.0001); nestin: F (3, 20) = 18.29, R^2^ = 0.7329, *p* < 0.0001) and vehicle group (Notch1: (F (3, 20) = 17.34, R^2^ = 0.7223, *p* = 0.0423); nestin: F (3, 20) = 18.29, R^2^ = 0.7329, *p =* 0.0168). This indicates that CA effectively reverses L-met-induced depletion while simultaneously promoting the expression of these markers (Figure 4).

### 3.4. Effect of CA on L-Met-Induced Apoptosis Protein Expression

**Figure 5:**
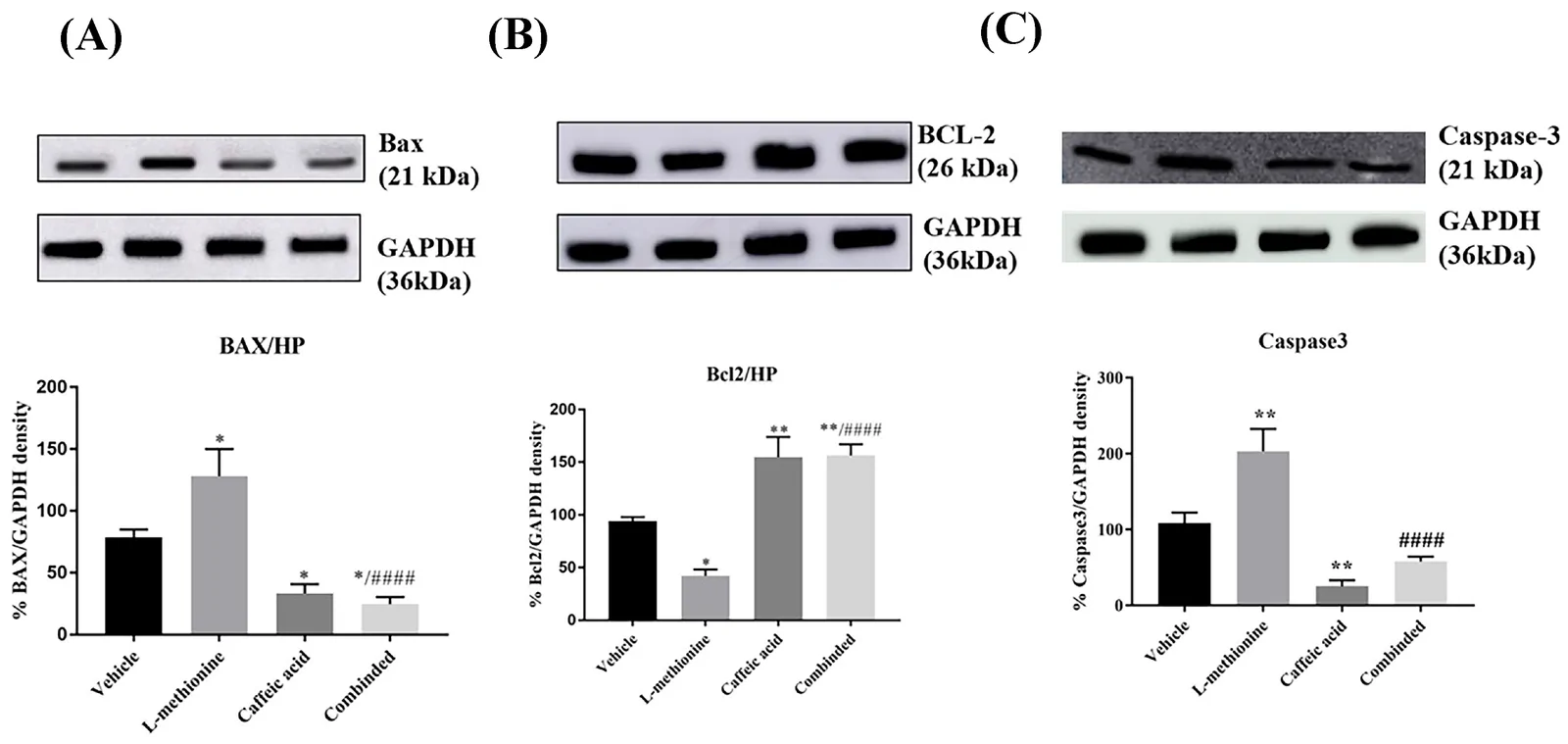
Protein expression levels in the hippocampal tissue: (**A**) Bax, (**B**) Bcl-2, and (**C**) caspase-3. Data are compared with the vehicle group (* *p* < 0.05, ** *p* < 0.01) and the L-met group (^####^ *p* < 0.0001).

The administration of L-met significantly upregulated expression of the pro-apoptotic protein Bax compared with the vehicle group (F (3, 20) = 15.03, R^2^ = 0.6928, *p* = 0.0307). Conversely, in the combined group, Bax expression was significantly downregulated compared to that in the L-met group (F (3, 20) = 15.03, R^2^ = 0.6928, *p* < 0.0001). Notably, treatment with CA alone significantly reduced Bax expression compared with the vehicle group (F (3, 20) = 15.03, R^2^ = 0.6928, *p* = 0.0493), demonstrating its capacity to suppress pro-apoptotic expression. Furthermore, the expression of the anti-apoptotic protein Bcl-2 was significantly decreased in the L-met group compared with the vehicle and combined groups (F (3, 20) = 22.64, R^2^ = 0.7725, both *p* < 0.0001). Concurrently, the expression of Bcl-2 reflects its established role in suppressing apoptosis. Furthermore, the group treated with CA exhibited a significant increase in Bcl-2 expression compared with the vehicle group (F (3, 20) = 22.64, R^2^ = 0.7725, *p* < 0.0497), suggesting that CA actively promotes the anti-apoptotic marker expression. Caspase-3 expression mirrored the patterns observed in mitochondrial apoptotic markers, showing a consistent trend across all experimental groups. Western blot analysis revealed that caspase-3 expression in the L-met-treated group was significantly elevated compared with the vehicle group (F (3, 20) = 20.57, R^2^ = 0.7552, *p* = 0.0025). Conversely, CA co-administration in the L-met group significantly reduced caspase-3 expression relative to the L-met group (F (3, 20) = 20.57, R^2^ = 0.7552, *p* < 0.0001). Furthermore, CA treatment alone induced a significant downregulation of caspase-3 expression compared with the vehicle group, confirming its neuroprotective role in attenuating apoptotic signaling (Figure 5).

## 4. Discussion

The present study demonstrates that L-met administration reduced the nestin-positive population, as evidenced by a marked decrease in the number of nestin-positive cells. A pivotal observation was the dynamic alteration in nestin-positive cells within the hippocampal DG. L-met administration significantly reduced nestin-positive cell density relative to the vehicle group, indicating that L-met induced metabolic disturbances impaired the maintenance and proliferation of hippocampal neural progenitor cells. By contrast, CA treatment expanded the nestin-positive cell population compared to the vehicle group, highlighting its endogenous neurogenic potential [21]. Co-administration of CA with L-met not only counteracted the toxic effects of L-met but also drove a robust recovery of progenitor cells [22]. Cell counts were significantly higher than in both the L-met and vehicle groups, suggesting that CA activates a compensatory pathway that mitigates metabolic stress-induced damage [23]. Collectively, these findings demonstrate that CA is a potent neuroprotective agent that mitigates L-met-induced neurotoxicity and supports neural progenitor cell survival. Furthermore, our nestin expression provides a mechanistic link that complements previous observations of impaired hippocampal neurogenesis under HHcy conditions. Previous studies using identical dosing regimens (CA 40 mg/kg; L-met 1.7 g/kg; oral gavage) demonstrated that CA potential regulates the neurogenic niche, counteracting L-met-induced metabolic insult [15]. The parallel modulation of nestin, Ki-67, and BrdU/NeuN is consistent with our previous findings using the same protocol [15]. This structural preservation, reflected in nestin expression, underlies Sox-2-mediated regulatory stability. Together with synchronized changes in Sox-2 and DCX, the nestin pattern reveals a kinetic bridge within the neurogenic lineage. While Sox-2 governs stemness identity, nestin provides the necessary cytoskeletal plasticity for progenitor cells to transition from a quiescent state to an active proliferative phase, a process frequently disrupted by neurogenic arrest under L-met accumulation. Consequently, in our previous study using the same L-met (1.7 g/kg) and CA (40 mg/kg) dosing regimens under the same experimental conditions, we reported parallel modulation of Sox-2, DCX, Ki-67, and BrdU/NeuN expression, indicating that the CA-mediated preservation of the neurogenic niche extends from the stem/progenitor stage through proliferative capacity to neuronal commitment [7,15]. Collectively, these findings indicate that CA preserves the neurogenic niche, consistent with our earlier tracking of these downstream markers.

Adult neurogenesis within the SGZ of the hippocampal dentate gyrus is a tightly regulated process essential for cognitive function, and its impairment is increasingly linked to metabolic imbalances [24]. Previous studies have demonstrated that L-met, administered at 1.7 g/kg, acts as a potent disruptor of this process, inducing cell cycle arrest—reflected by increased p21-positive cell expression—and consequently reducing both overall cell proliferation and the population of Sox-2-positive neural stem cells [24,25]. Our findings further extend this pathological model by revealing a significant reduction in nestin-positive cells following L-met exposure. The cell depletion is highly consistent with the observed volumetric reductions in both the total DG and SGZ. These findings suggest that L-met-induced reductions in nestin-positive cells and hippocampal volume reflect not only molecular disruption but also architectural decline within the neurogenic niche, linking molecular alterations to structural integrity. Our findings elucidate how L-met-induced HHcy leads to progressive neurogenic atrophy. Notably, in addition to preventing these cellular alterations, CA effectively mitigates volume loss in both the DG and SGZ. Our results are further corroborated by previous studies on chrysin, a structurally related flavonoid known for its potent neuroprotective and antioxidant properties [26]. Collectively, these findings reinforce the potential of CA to attenuate L-methionine-induced reductions in DG and GCL volume in this experimental model. The molecular interplay between nestin and Notch1 constitutes a critical regulatory circuit in the maintenance of hippocampal neural stem cell homeostasis [27]. Our Western blot findings indicate that the downregulation of Notch1 under HHcy conditions is intrinsically linked to a concurrent decrease in nestin expression. Specifically, Notch1 signaling functions as a molecular switch that maintains the nestin-positive state, preserving the pool of undifferentiated, highly proliferative neural progenitor cells [28]. Mechanistically, previous studies suggest that upon activation, Notch1 translocates to the nucleus to interact with transcriptional complexes, suppressing pro-apoptotic signaling and preventing premature differentiation to maintain this undifferentiated phenotype [29]. Notch1 signaling preserves the self-renewal capacity of the NSC pool; thus, its downregulation under HHcy conditions compromises this regulatory architecture. The resulting downregulation of Notch1 removes the requisite stimulus for maintaining nestin expression, triggering a cascade that forces these progenitors to prematurely exit the cell cycle [30]. This shift from a proliferative to a senescence-like pathway compromises the structural integrity of the neurogenic niche and significantly diminishes its long-term regenerative capacity [31]. The impairment of the Notch1–nestin axis is primarily driven by HHcy, which is initiated by excess L-met. Elevated levels of L-met disrupt cellular redox balance, leading to the accumulation of reactive oxygen species (ROS) and the activation of pro-inflammatory cytokines within the hippocampal niche [32]. This oxidative environment acts as a potent negative regulator of Notch1 signaling, specifically impeding proteolytic cleavage and nuclear translocation of the Notch intracellular domain, processes essential for nestin transcriptional activation and neural stem cell self-renewal [31]. In contrast, CA functions as a strategic pharmacological modulator, restoring this regulatory architecture by scavenging ROS and mitigating the neuroinflammatory response induced by L-met [7]. CA prevents oxidative degradation of the Notch1 signaling machinery and stabilizes nestin expression by fostering a more favorable neurogenic microenvironment, thereby rescuing NSCs from premature senescence or terminal differentiation. Collectively, these findings demonstrate CA’s potential to mitigate molecular mechanisms underlying HHcy-associated neurocognitive decline, consistent with observations in other experimental models [33,34].

The observed modulation of apoptosis markers within the hippocampal dentate gyrus may help explain the neurogenic impairment induced by L-met. In the SGZ, the primary site of adult hippocampal neurogenesis, the maintenance of the neural progenitor pool is highly sensitive to the mitochondrial apoptotic threshold. Previous studies have shown that L-met administration triggers mitochondrial-mediated apoptosis by elevating oxidative stress and increasing the Bax/Bcl-2 balance [35]. According to this proposed mechanism, the resulting metabolic insult induces endoplasmic reticulum stress, leading to the accumulation of unfolded proteins that suppress Bcl-2, directly promote Bax transcription, and facilitate the release of cytochrome c into the cytosol. This cascade culminates in the activation of the executioner caspase-3, creating a feedback loop that accelerates cellular death [36]. Although the present study did not directly assess mitochondrial translocation, cytochrome c release, or caspase-3 cleavage, this proposed molecular sequence may plausibly contribute to the depletion of the progenitor pool in the SGZ, thereby explaining the observed reduction in nestin expression. The restorative efficacy of CA against L-met-induced neurotoxicity is fundamentally anchored in its ability to modulate the mitochondrial apoptotic threshold within the hippocampal dentate gyrus. Hcy induces a deleterious shift in Bax characterized by upregulation of the pro-apoptotic protein [37]. In the present study, co-administration of CA with L-met attenuated pro-apoptotic signaling. The CA-treated group exhibited a statistically significant reduction in Bax and caspase-3 expression, along with a notable increase in Bcl-2 expression, compared to the L-met group [38]. Taken together, these findings suggest that CA counteracts L-methionine-induced changes in Bax, Bcl-2, and caspase-3 expression in the hippocampal dentate gyrus. Although not direct proof, these findings are consistent with a role for CA in preserving mitochondrial homeostasis and attenuating the terminal caspase-3 cascade. The use of a mild, non-fractionating lysis protocol means that the mitochondria-associated subpopulation of Bax and Bcl-2 may not be fully represented in the fraction analyzed, which can be affected by nucleic acid and detergent content in crude tissue homogenates. The literature suggests that the protective effects of CA may extend beyond direct ROS scavenging. For example, L-met-induced metabolic dysregulation has been linked to phosphorylation of c-Jun N-terminal kinases (JNK) and p38 mitogen-activated protein kinase, pathways implicated in the transcriptional upregulation of Bax and destabilization of Bcl-2 [39]. It has been proposed that polyphenols such as CA may suppress these stress-activated protein kinases; however, this was not assessed in the present study. Furthermore, the reduction in caspase-3 expression observed in the combined group is consistent with modulation of the apoptotic cascade upstream or downstream of caspase-3 itself [38]. Limitations of the present study include the absence of direct evaluation of inflammatory responses. These aspects will be addressed in future investigations, including the use of subcellular fractionation and validated colorimetric protein assays, to elucidate the underlying neuroprotective mechanisms better.

## 5. Conclusions

This study demonstrates that CA attenuates L-met-induced hippocampal neurotoxicity in a rat model. CA administration preserved DG and SGZ volumes and maintained nestin expression, confirmed by both immunofluorescence and Western blot analysis, indicating protection of the neural progenitor cell pool from L-met-induced atrophy. This protection was accompanied by changes in Notch1 expression and by concurrent modulation of apoptosis-related protein expression, including downregulation of the pro-apoptotic proteins Bax and caspase-3 and upregulation of the anti-apoptotic protein Bcl-2 in the CA-treated groups compared with the L-met group.

## Data Availability

The data that support the findings of this study are available within the article and its Supplementary Material.

## Supplementary Materials

The following supporting information can be downloaded at: https://www.mdpi.com/article/10.3390/biology15181615/s1, File S1: Original Western blot for three repeats.

## Abbreviations

The following abbreviations are used in this manuscript:

°C	degrees Celsius
CA	caffeic acid
DCX	doublecortin
DG	dentate gyrus
GCL	granular cell layer
L-met	L-methionine
ROS	reactive oxygen species
SGZ	subgranular zone
SAH	S-adenosylhomocysteine
