# Supplementary material for: Caffeic Acid Mitigates L-Methionine-Induced Impairment of Hippocampal Neural Stem Cells Through Anti-Apoptotic Signaling in Adult Rats
Source: Biology (Basel). 2026 Sep 14;15(18):1615. doi: 10.3390/biology15181615 (PMC13604278; doi:10.3390/biology15181615)

Caffeic acid mitigates L-methionine-induced impairment of hippocampal neural stem cells through anti-apoptotic signaling in adult rats

Oabnithi Dornlakorn[1,2], Nataya Sritawan[1,2], Ram Prajit[1,2], Apiwat Sirichoat[1,2], Peter Wigmore[3] and Jariya Umka Welbat[1,2,*]

1 Department of Anatomy, Faculty of Medicine, Khon Kaen University, Khon Kaen 40002, Thailand; oabnithiatlakorn@kkumail.com (O.D.); natasr@kku.ac.th (N.S.); rampra@kku.ac.th (R.P.); apiwsi@kku.ac.th (A.S.)

2 Neurogenesis Research Group, Faculty of Medicine, Khon Kaen University, Khon Kaen 40002, Thailand

3 School of Life Sciences, Medical School, Queen's Medical Centre, The University of Nottingham, Nottingham NG7 2RD, UK; peter.wigmore@nottingham.ac.uk

* Correspondence: jariya@kku.ac.th

Original Western blot for three repeats

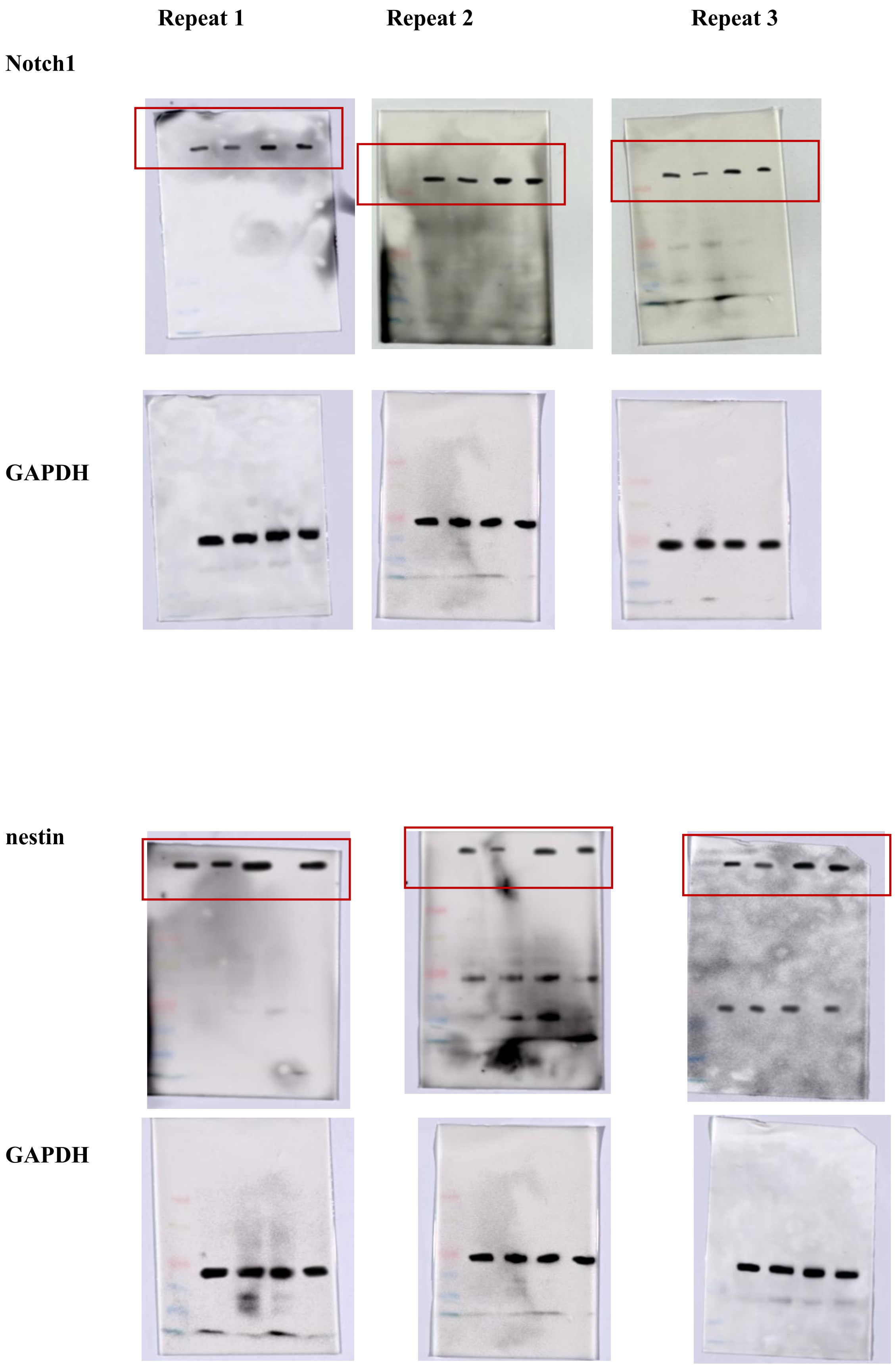

Repeat 1 **Repeat 2** **Repeat 3**

Bax

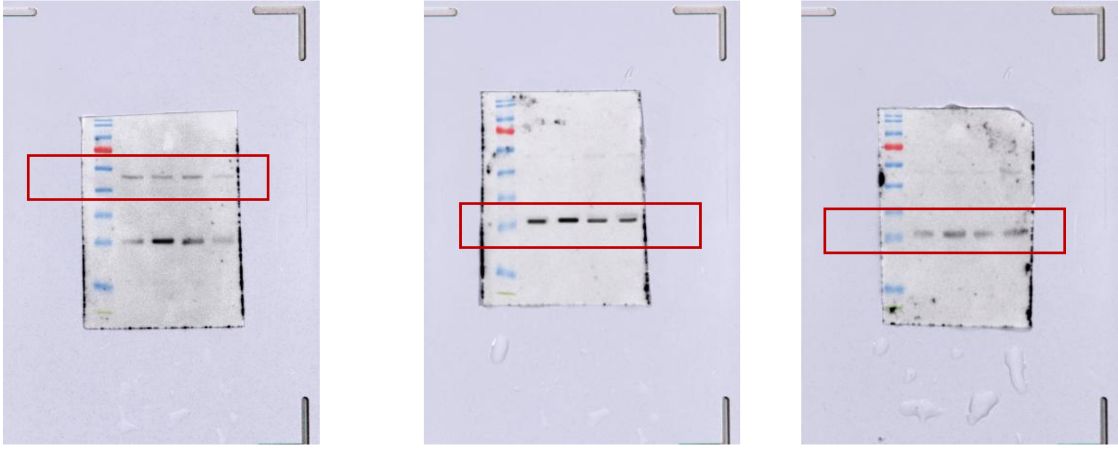

GAPDH

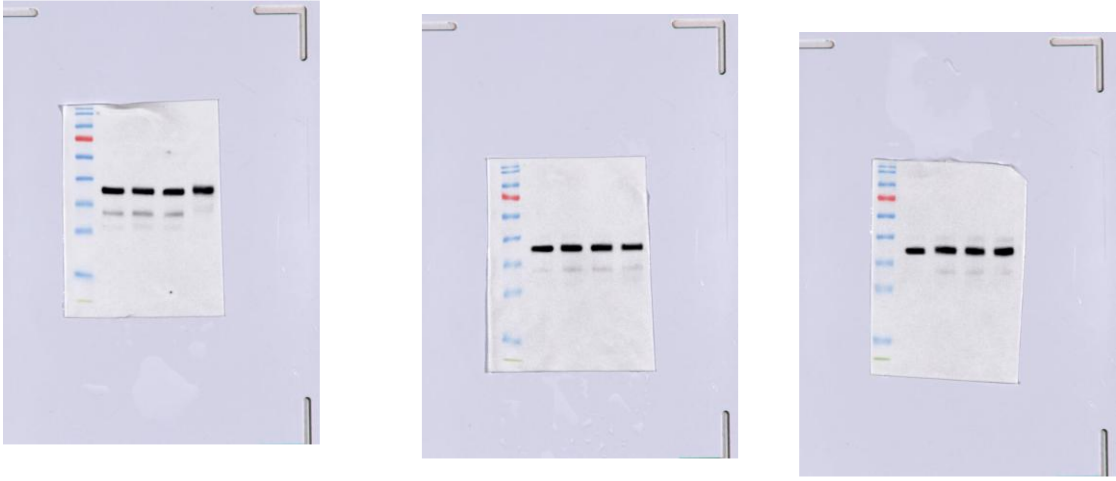

BCL-2

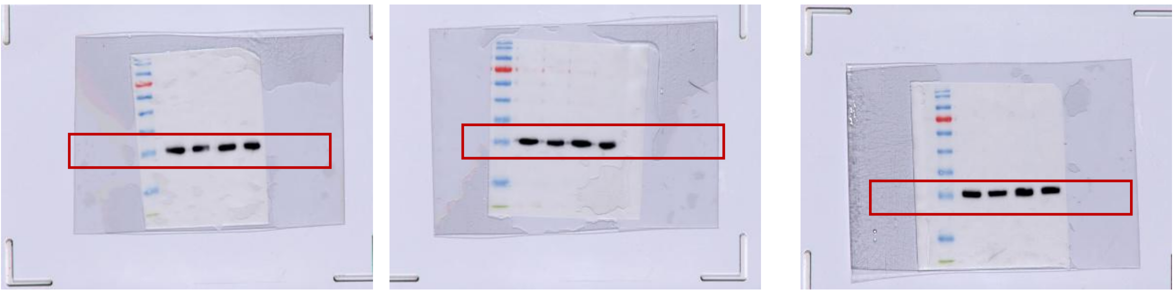

GAPDH

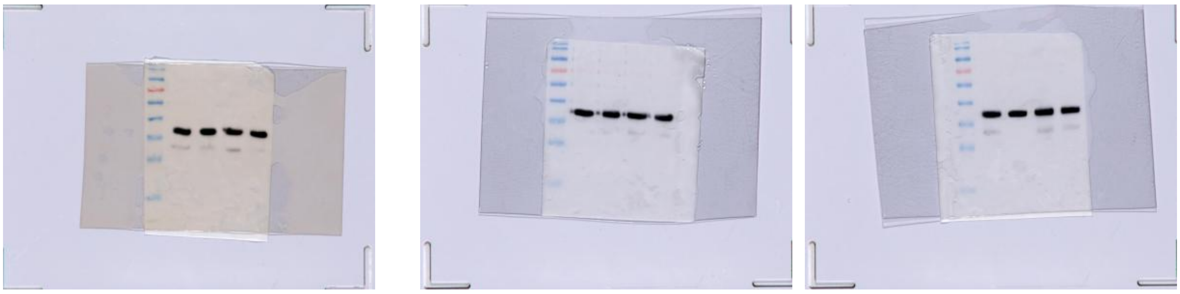

Repeat 1 **Repeat 2** **Repeat 3**

Caspase-3

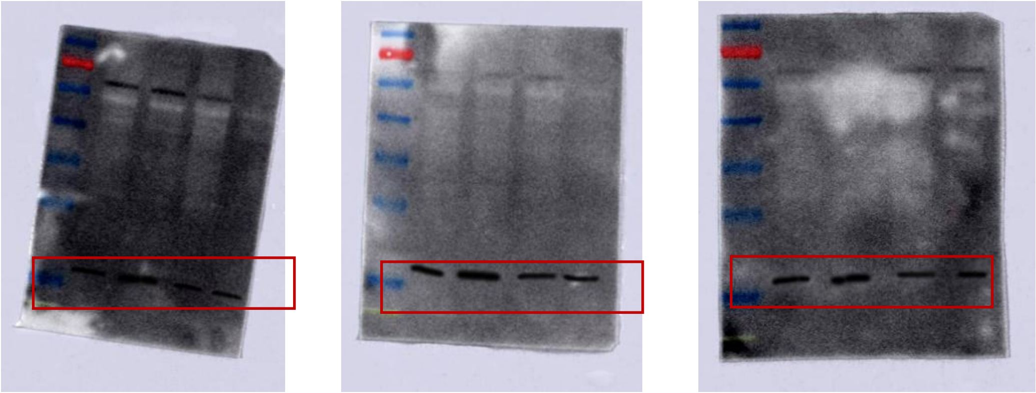

GAPDH

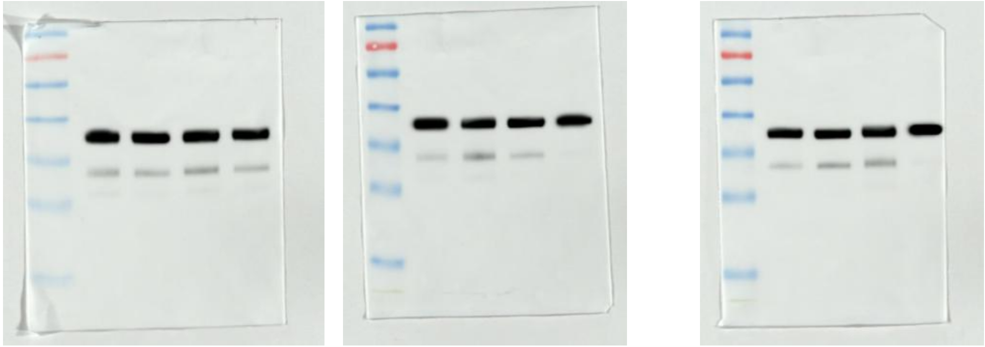

Supplement: Supplementary file 1 [file biology-15-01615-s001.zip › biology-4509188-supplementary.pdf]
